# Nutrition Facts in the Over-Eighty Population: A Narrative Review

**DOI:** 10.3390/nu17172740

**Published:** 2025-08-24

**Authors:** Paolo Riccio, Emilio Jirillo

**Affiliations:** 1 Independent Researcher, 70126 Bari, Italy; 2 Interdisciplinary Department of Medicine, Medical School, University of Bari, 70100 Bari, Italy; emilio.jirillo@uniba.it

**Keywords:** aging, sarcopenia, minerals, dietary supplements

## Abstract

*Background*: For the first time, humanity is facing the worldwide challenge of global population aging over 80 years. As individuals age, energy acquisition and metabolism undergo significant changes, leading to a progressive decline in energy intake, absorption, and utilization. These changes contribute to malnutrition, loss of muscle mass, frailty, hormonal decline, mineral depletion, and impaired hydration, all of which increase the risk of morbidity and decrease quality of life. In addition, as life expectancy increases, advanced age often brings a gradual loss of autonomy, mirroring early-life dependency. *Objectives*: Addressing this age shift requires targeted interventions to support the wellness of the growing very elderly population. This review provides an overview of healthy aging through an integrated approach that includes nutritional intervention, lifestyle modifications, and targeted supplementation to support functional independence and overall well-being in older adults. The guiding principle is that longevity matters less than aging well.

## 1. Introduction

Old age has been addressed since antiquity, even at times when it barely existed. In ancient Rome, approximately 2000 years ago, famous figures such as Cicero, Seneca, and Publius Terentius Afro wrote about old age.

Due to high infant mortality, at that time, average life expectancy was approximately 25–30 years. Most people lived no more than 40–60 years and reaching 80 years was an exceptional occurrence. Despite its rarity, old age was debated in contrasting terms: Publius Terentius Afro described it as “*Senectus ipsa morbus est*” (“old age itself is a disease”), whereas Cicero valued its virtues, and Seneca argued that, when adequately supported, it could represent a positive and even essential stage for the benefit of the community.

### 1.1. Aging: A Natural Phase of Life

All living organisms are subject to spatial and temporal constraints that define their size range and lifespan. As in the time of Publius Terentius Afro in ancient Rome, some researchers have proposed that old age should be considered a disease [1,2]. However, aging may be more appropriately regarded as a natural phase of life [3,4], a biological process that becomes pathological only when it occurs significantly earlier than the population average, and not as a result of accidental causes.

Aging is intrinsic to biological systems because every living organism can be regarded as a form of condensed energy. Maintaining this state requires continuous energy input to preserve structural integrity and to sustain maintenance and repair mechanisms. Within our space–time framework, the ability to acquire energy is time-limited, restricting the longevity of biological systems to a finite duration. This limitation is not dictated by specific genetic programs for aging but results from the gradual loss of molecular fidelity over time [5,6]. Repair and renewal processes are inherently imperfect and never completely efficient. Consequently, indefinite survival is incompatible with the nature of life. Lifespan is ultimately determined by the balance between the frequency of structural damage and the efficiency of maintenance mechanisms at the cell, tissue, and organ levels (Table 1).

Therefore, aging results from the progressive inefficiency of maintenance and repair systems [6]. This means that although aging can be modulated, it remains fundamentally irreversible.

Maintenance is sustained by the continuous intake of exogenous molecules—primarily through feeding. Eating is not merely a matter of caloric intake; it also ensures the continuous renewal and structural preservation of the organism. The nutrients we ingest originate from other forms of life—distinct from us in their structure and in their polymeric compounds but identical to us in their monomeric fundamental units (e.g., glucose, amino acids, and fatty acids). These monomers are transformed into our own molecular constituents—thus, what we call maintenance or turnover can be interpreted as a transient propagation of life through the act of feeding ourselves.

### 1.2. The Challenge of Global Population Aging

In addition to the ongoing rise in the global population and the challenges posed by climate change, humanity is now facing, for the first time, the issue of the growing number of people aging over the 80 years. Currently, individuals over the age of 80 constitute approximately 1.6% of the global population, and this percentage could increase to 4% by 2050 [7,8,9] (Table 2).

Furthermore, according to the European Commission, the proportion of individuals aged 80 and over in the European Union increased from 3.7% in 2003 to 6.0% in 2023. Among EU member states, Italy had the highest median age, indicating a large presence of elderly individuals and a substantial aging population [10,11].

Countries like Mexico, Israel, the Philippines, and India are characterized by younger populations, in contrast to Japan, which has one of the highest proportions of elderly individuals worldwide [9] (Table 2).

## 2. The Gastrointestinal System in Old Age

In individuals over 80 years of age, the body undergoes a marked physiological decline. While cognitive function is often mostly preserved—excluding those with neurodegenerative diseases—the gastrointestinal system, being highly susceptible to cumulative wear and tear, is among the first to experience structural and functional deterioration.

Gastrointestinal dysfunction plays a pivotal role in aging, as it is essential for nutrient absorption and energy acquisition. A decline in digestive efficiency compromises the extraction of macronutrients and micronutrients, leading to energy deficits that impair metabolism and immune defense. These deficiencies may severely compromise the individual’s ability to maintain independence and quality of life.

Common digestive impairments in those over 80 years include the following:

(i) Reduced masticatory capacity [12,13], particularly when dental interventions are no longer feasible due to old age [14];
(ii) Decreased saliva production [15];
(iii) Reduced gastric acid and digestive enzyme secretion [16];
(iv) Impaired intestinal peristalsis [17];
(v) Slower gastric emptying [18], which may increase the risk of gastroesophageal reflux;
(vi) Reduced gut microbiota diversity [19].

The risk of malnutrition is further exacerbated by diminished senses of smell and taste, as well as by social isolation, dietary restrictions, and constipation, all of which may lead to reduced appetite and inadequate nutritional intake [20].

In old age, dietary adjustments should account for these physiological changes. To promote adequate intake and optimize digestive efficiency, it would be best to favor easily digestible and easily masticable foods, such as yogurt, vegetable or legume purees, creamy soups, smoothies, and juices. From a general nutritional standpoint, adherence to the principles of the Mediterranean diet, with extra-virgin olive oil as the primary source of dietary fat, is recommended [21].

Beyond overcoming masticable difficulties, the choice of soft over solid foods also has energetic implications. Solid meals require more energy for mastication, digestion, and absorption compared to equivalent soft meals. For example, a 100 kcal solid meal may require an energy expenditure of approximately 10–15 kcal, whereas a soft or liquid meal of the same caloric value may require only 5–10 kcal. This difference can be significant in preserving energy balance in the elderly.

## 3. Immune Aging

Aging is characterized by a progressive loss of immune function. In fact, there is evidence that hemopoietic stem cells, which differentiate into myeloid cells [granulocytes, monocytes, and dendritic cells (DCs)] and lymphoid cells [T lymphocytes, B lymphocytes, and natural killer (NK) cells)], undergo a progressive decrease in density and regeneration potential, thus contributing to age-dependent immune deficiency [22]. On the other hand, the thymus (where T lymphocytes differentiate) starts declining in early childhood, with a progressive loss of its architecture [23].

The immune system consists of two major components, innate immunity and adaptive immunity, which are both compromised with aging [24]. This implies an impaired immune responsiveness against microorganisms, cancer cells, and autoreactive clones, thus determining the condition of immune senescence [25]. “Inflammaging” is the term used for low-grade chronic inflammation, which increases with advancing age, as a result of constant antigenic exposure [26].

As far as alterations of innate immunity in human aging are concerned, a decrease in neutrophils after 65 years has been reported [27]. Furthermore, neutrophils have been shown to cause telomere damage via release of reactive oxygen species (ROS), thus inducing senescence [28]. Aged monocytes exhibit impaired phagocytosis, with increased levels of Tumor Necrosis Factor (TNF)-alpha release and decreased levels of interleukin (IL)-1 and IL-6 production [29], while dendritic cells (DCs), as antigen-presenting cells, are less tolerogenic, with reduced migration and antigen presentation [30].

Regarding adaptive immunity in aging, a decline in the production of naïve T cells and a loss of diversity in the peripheral T cell repertoire have been documented [26]. Furthermore, aged T cells lose CD28 expression, with impaired responsiveness to antigens, while CD8+ T cytotoxic cells and T regulatory (Treg) cells undergo a reduction in their anti-cancer activity and suppressive functions, respectively [31]. B cell output from the bone marrow is reduced, with a loss of class switching and somatic hypermutation for the formation of high-affinity antigen-specific antibodies [32]. In aged people, the ability of NK cells to lyse specific targets is lost, with decreased perforin release [33]. On the contrary, aging does not affect NK-mediated antibody-dependent cytotoxicity [34].

## 4. Gut Microbiota in Aging and Its Influence on Immune Response

The gut microbiota represents the largest microbial community in the human body, comprising bacteria, fungi, and viruses [35,36]. During aging, its composition and functionality undergo significant alterations, influenced by dietary habits, pharmacological treatments, and the age-related decline in immune competence. These changes include a reduction in microbial diversity, loss of beneficial taxa, and a relative enrichment of potentially pathogenic species [37].

In younger adults, the gut microbiota is typically dominated by *Firmicutes* and *Bacteroidetes*, together accounting for over 90% of colonic bacterial taxa. The *Firmicutes/Bacteroidetes* (F/B) ratio is often used as an ecological index; however, its variation with age is inconsistent. Some authors report a reduced F/B ratio in the elderly, reflecting a relative increase in *Bacteroidetes* [38,39], while others find the opposite trend or no significant change [40,41]. Such discrepancies may arise from differences in diet, lifestyle, genetic background, health status, and sequencing methodologies. Regardless of type of shifts, changes in these dominant *phila* are often associated with decreased production of short-chain fatty acids (SCFAs), such as acetate, propionate, and butyrate, which are crucial for intestinal barrier integrity, immune regulation, and host metabolic function.

Aging-associated dysbiosis is often linked to inflammaging, the chronic low-grade inflammatory state associated with increased intestinal permeability (“leaky gut”). This facilitates the translocation of microbial products, undigested food particles, and endotoxins into the systemic circulation, contributing to chronic inflammation, metabolic dysfunction, and age-related diseases.

Aging is specifically associated with a decline in beneficial butyrate-producing taxa, such as *Faecalibacterium prausnitzii*, *Eubacterium rectale*, *Clostridium septum*, and *Roseburia* spp., compromising epithelial barrier function and immune regulation [42,43]. Conversely, *Proteobacteria* expand, releasing immunostimulatory lipopolysaccharides (LPSs) that promote cytotoxicity, immune activation [44], and immune senescence [45]. Notably, *Bacteroides*-derived propionate has been shown to enhance resistance to Salmonella spp. in murine models [46]. More broadly, the gut microbiota confers colonization resistance by limiting pathogen overgrowth through competition for nutrients and niche exclusion [47].

Microbiota-derived metabolites also exert direct immunomodulatory effects: for example, butyrate promotes the differentiation of colonic Treg cells. In adult humans, fecal microbiota transplantation (FMT) has been shown to restore immune homeostasis following hematopoietic stem cell transplantation [48].

Reduced SCFA production and compromised barrier integrity further promote microbial translocation and systemic inflammation, which are key contributors to chronic age-related diseases [49]. Moreover, decreased *Lactobacillus*, *Akkermansia*, and certain *Bacteroidetes* spp. have been linked to weakened tight junctions and reduced mucus production [50], whereas overgrowth of pathobionts such as *Clostridium difficile* disrupts epithelial integrity and mucus layer homeostasis [51].

## 5. Mitochondrial Dysfunction and Cellular Senescence

Mitochondria are central regulators of cellular senescence, contributing to its initiation and progression through metabolic and redox dysregulation. During oxidative phosphorylation, mitochondria generate reactive oxygen species (ROS) as byproducts. While physiological antioxidant systems normally buffer ROS, dysfunctional mitochondria produce excessive ROS and display impaired repair responses, leading to oxidative damage of DNA, proteins, and lipids and triggering senescence [4].

Mitochondrial DNA (mtDNA) is particularly vulnerable to oxidative lesions due to its proximity to the electron transport chain and its limited repair capacity. The accumulation of mtDNA mutations further impairs respiratory chain function, reinforcing mitochondrial dysfunction in a self-amplifying cycle [4]. In parallel, aging disrupts mitochondrial dynamics, resulting in decreased ATP synthesis [52].

Aging also compromises mitophagy, the selective autophagic removal of damaged mitochondria. The accumulation of dysfunctional mitochondria exacerbates redox stress and promotes senescence [53].

Mitochondrial dysfunction acts in multiple senescence pathways [54]. Targeted strategies to restore mitochondrial integrity, such as mitochondria-directed antioxidants, mitophagy activators, and caloric restriction mimetics, have demonstrated efficacy in reducing senescence markers and increasing health span in preclinical models [55,56,57]. Overall, mitochondria play an active role in cellular aging, representing plausible therapeutic targets for mitigating senescence [58,59].

## 6. Deficiency of Mineral Salts in the Elderly

In individuals aged 80 years and over, physiological aging increases susceptibility to mineral deficiencies. The following overview summarizes the key biochemical roles of major minerals in this age group.

- Calcium, Phosphorus, and Magnesium: These minerals are vital for bone strength, neuromuscular conduction, enzyme activity, and blood coagulation. With advancing age, osteopenia and osteoporosis are common; inadequate calcium and magnesium support can accelerate bone demineralization and raise fracture risk [60].
- Potassium and Sodium are essential for fluid balance, cardiac and neuromuscular excitability, and blood pressure regulation. People aged 80 and over are most sensitive to increased sodium and may benefit from lower-salt and higher-potassium diets to prevent hypertension, and cardiovascular and renal disease [61].
- Zinc is crucial for DNA synthesis, immune competence, wound healing, and enzymatic reactions. Old people often do not meet recommended zinc intake. Lower levels of zinc exacerbate vulnerability to infections [62].
- Selenium and Copper support antioxidant defense, erythropoiesis, and collagen synthesis. Insufficiency of selenium, zinc, iodine, and copper has been observed among older adults [63].
- Iron maintains hemoglobin levels and physical performance; deficiency in the elderly impairs functional capacity [64].
- Selenium and Magnesium have been linked to reduced sarcopenia. Selenium, together with Calcium intake, is significantly associated with muscle mass, whereas together with Magnesium intake, it is significantly associated with physical performance [65].

### Clinical and Nutritional Considerations

○ A varied diet based on DASH (Dietary Approaches to Stop Hypertension) recommendations [61] or on a Mediterranean diet with preferential use of extra-virgin olive oil generally provides adequate mineral intake. However, intestinal malabsorption may still impair mineral status.
○ Given the risk of deficiencies in old age, both periodic screening and, when necessary, supplementation under clinical supervision are recommended.

In summary, for individuals aged 80 years and over, ensuring adequate mineral intake is critical to maintaining systemic balance, preventing frailty and preserving physical and cognitive function. Nutritional surveillance and individualized interventions are essential to mitigate the clinical impact of deficiencies and support healthy aging and functional independence.

## 7. Sarcopenia in the Elderly: Prevention and Management

One of the main conditions to avoid or manage in old age is sarcopenia—the progressive loss of skeletal muscle mass, strength, and function.

Malnutrition accelerates muscle loss [66,67]. Age-related muscle decline results from hormonal changes, reduced physical activity, and chronic low-grade inflammation, but targeted nutritional strategies, exercise, and medical interventions can attenuate these effects. Loss of muscle strength leading to frailty is a key limiting factor for maintaining independence until the end of life. Nutritional interventions can not only prevent but also partially reverse sarcopenia [67,68,69]. A high-protein diet and regular physical activity are central components of prevention and treatment.

### Exercise and Physical Activity

Regular strength and resistance training is the most effective intervention against sarcopenia. Balance training (Tai Chi, yoga, and stability exercises) helps prevent frailty [70,71]. Aerobic activities, such as walking, performed daily for 30–45 min, support cardiovascular health, mobility, and endurance. Minimizing sedentary behavior is equally important; prolonged sitting accelerates muscle atrophy, while everyday activities such as housework, gardening, or short walks promote mobility.

Sarcopenia markedly increases the risk of frailty, falls, and loss of independence. A combined approach—including adequate nutrition, resistance training, and regular medical monitoring—enhances muscle health, quality of life, and longevity. Maintaining an active lifestyle, supported by individualized supplementation, and routine check-ups is essential to preserve muscle strength and functional capacity in older adults.

The etiology of sarcopenia in very old age is multifactorial. Adequate mineral intake is associated with better physical performance and a reduced risk of sarcopenia, reinforcing the need for integrated nutritional and physical activity interventions.

## 8. Hormonal Therapy

As with many bioactive molecules, hormone production declines with age [72,73,74] This reduction is a major contributor to the structural and functional deterioration observed in aging. However, such decline should be regarded as a physiological process and, therefore, as part of normal aging. Acceptance of reduced hormone levels does not preclude intervention when clinically indicated, but in extreme old age, hormone administration should only be performed under specialist supervision and after confirming a documented deficiency, given the potential contraindications and risks.

Examples of hormone replacement strategies include the following:

- Testosterone Replacement Therapy [75].
- Estrogen and Progesterone Therapy (for postmenopausal women).
- Dehydroepiandrosterone (DHEA) supplementation [76].

DHEA—a precursor of both testosterone and estrogen—has been reported to exert immunostimulatory, anti-diabetic, anti-atherosclerotic, neuroprotective, anti-obesity, and anti-osteoporosis effects [76].

- Thyroid Hormone Replacement (Hypothyroidism); Growth Hormone (GH) Therapy; Insulin-like Growth Factor 1 (IGF-1)

As with other hormones, GH and IGF-1 levels decline with age [77,78]. IGF-1 helps prevent sarcopenia and enhances muscle repair and recovery after injury. It also influences glucose metabolism, insulin sensitivity, and fat distribution; contributes to energy balance and cellular repair mechanisms; and supports neuronal growth and protection, potentially improving memory and cognitive function.

In conclusion, hormone therapy in the elderly can provide meaningful benefits but must be implemented only after a careful, individualized risk/benefit evaluation.

## 9. Urinary Tract Infection and Hydration

### 9.1. Lower Urinary Tract Infection

Aging predisposes to recurrent urinary tract infections (UTIs), frequently caused by uropathogenic *Escherichia coli* (UPEC) migrating from the intestine to the lower urinary tract [79]. This process is facilitated by age-associated gut dysbiosis-characterized by reduced microbial diversity, *E. coli* over-representation, and impaired intestinal barrier function. Recurrent UTIs in the elderly often involve genetically identical fecal and urinary isolates, suggesting an endogenous origin [79,80].

Microbiome-based interventions, such as *Lactobacillus rhamnosus* and *L. reuteri* supplementation, have demonstrated efficacy comparable to antibiotic prophylaxis without promoting antimicrobial resistance [81].

Fecal microbiota transplantation (FMT) has also proven effective, restoring microbial diversity and reducing symptomatic episodes [82]. From this perspective, UTIs in older adults may be viewed as a “gut-mediated disease”.

For the prevention of UTI, periodic urine culture testing is advisable.

### 9.2. Hydration Status

Hydration status is a critical yet overlooked aspect of geriatric nutrition. By the age of 80, total body water declines to 45–50% of body weight, compared to about 60% in younger adults [83], largely due to the loss of fat-free mass, particularly skeletal muscle [84].

Deydration—frequently underdiagnosed [85]—can negatively affect cognitive performance and exacerbate fatigue. Current recommendations suggest a daily fluid intake of ≥1.6 L for females and ≥2.0 L for males. Preventive strategies include ensuring water availability, consuming small amounts frequently, at regular intervals. Eventually, palatability can be enhanced with natural flavoring agents such as lemon juice, yogurt, or herbs. Moreover, adequate protein intake supports muscle mass preservation, while isotonic beverages are recommended to maintain electrolyte balance.

## 10. General Nutritional Strategies, Intermittent Fasting, and Supplementation in the Elderly

With advancing age, the human body undergoes significant physiological changes: our metabolism slows and energy requirements decrease. Our nutritional needs change. Maintaining a balanced eating style becomes essential for living a long, healthy life. Nutritional recommendations for older adults emphasize reducing carbohydrate intake while increasing protein and fiber. We need less energy and more bricks to repair tissues.

For those relying on liquid meals, increasing meal frequency beyond three per day may help to achieve caloric adequacy. Sugar and gluten consumption should be minimized. The same for hydrogenated fats and processed foods. Monounsaturated fats (extra-virgin olive oil) and omega-3 polyunsaturated fats (oily fish) are recommended. Whole grains and flours should replace refined products, and animal-derived foods should be consumed only occasionally. Legumes, often under-consumed, should be included daily.

A diet rich in vegetables and legumes, with moderate fruit intake, is preferable. Vegetables are preferable to fruit which, if very sugary, should be eaten in moderation. Foods should be fresh, seasonal, locally sourced, and organic.

With solid meals, each bite should be masticated for a long time. Each meal should be given the right amount of time and attention. Prolonged mastication causes the stomach to produce less ghrelin, the hormone that stimulates appetite. The intestine, on the other hand, generates more cholecystokinin, the hormone that controls the appetite center, and GLP-1, the hormone that lowers blood sugar. In other words: the more we masticate, the less we tend to eat; the less we eat, the better we feel.

Physical activity—such as walking, resistance training, dancing, or gardening—should be integrated into daily life, together with mindfulness practices to improve cognitive performance [86].

### 10.1. Intermittent Fasting

Throughout human evolution, intermittent food scarcity—due to hunting failures or agricultural limitations—was a recurrent condition. Consequently, intermittent fasting and episodic caloric restriction were common, in contrast to the modern Western lifestyle, which is characterized by continuous food availability [87].

Evidence suggests that intermittent fasting and/or caloric restriction may confer health benefits, including enhanced intestinal barrier integrity, increased microbial diversity, and attenuation of gut inflammation [88,89,90,91,92,93]. Periods of fasting provide physiological rest for the gastrointestinal tract, potentially supporting long-term intestinal health.

Time-restricted feeding (12–16 h fasting windows) is a practical approach: Daily caloric intake is consumed within an 8–12 h period, with only non-caloric fluids thereafter. Advancing the last meal to early evening can improve intermittent fasting.

### 10.2. Supplementation

In addition to dietary strategies, sustained supplementation under qualified medical supervision can play a critical role in maintaining health in the elderly. Among micronutrients, vitamin D supplementation is a priority. An intake of 5000 I.U./day is recommended to maintain serum concentrations within the optimal range of 40–60 ng/mL [94]. Co-administration with vitamin A is advised, as the receptors of both vitamins act cooperatively in regulating gene expression, upon ligand binding [95].

Additional vitamins include vitamin B12 and vitamin C. In many cases, the use of a broad-spectrum multivitamin complex may be preferable to ensure adequate coverage of essential micronutrients.

Given the high prevalence of sleep disturbances in older adults, melatonin supplementation warrants consideration. There are several similarities between melatonin and vitamin D: both act as hormones, possess immunomodulatory and anti-inflammatory properties, are present in the skin, and respond to environmental light cycles—vitamin D to sunlight, melatonin to darkness [96,97]. Thus, the two molecules seem to have overlapping functions and complementary activities related to light exposure. Their combined use may provide synergistic benefits. Endogenous levels of both decline with age [98,99].

Other recommended supplements include the following:

(1) Essential amino acids, to support protein synthesis;
(2) Complex carbohydrates such as inulin, for prebiotic effects;
(3) Probiotics, to maintain microbiota balance [100];
(4) Butyrate, to preserve or restore intestinal barrier integrity.

For neuroprotection, phospholipids such as L-alpha-glycerophosphorylethanolamine may be beneficial, particularly in formulations combined with plant extracts, coenzyme Q10, and a mixture of antioxidants [101]. Among antioxidants, the modified polyphenol *polydatin* (resveratrol glucoside) is of particular interest for its high bioavailability.

To prevent or counteract sarcopenia, supplementation with L-leucine, omega-3 fatty acids, and *Lactobacillus paracasei* (OLEP) has been classified as food for special medical purposes, for the dietary management of sarcopenia, and for the maintenance and/or recovery of muscle strength and mass [102].

## 11. Conclusions

This review focuses on individuals over 80 years of age who are free from disease. Given the heterogeneity of this population, a multidimensional and individualized approach is essential to preserve their quality of life. The primary objective should be to ensure that people over 80 enjoy a dignified and as independent as possible old age.

### Actions to Be Taken

To achieve this goal, a dedicated task force on aging should be established, and special geriatric care units should be created to address the specific needs of this population. Preventive strategies to reduce frailty—including regular physical activity, adequate nutritional support, and vitamin D supplementation—are strongly recommended to maintain functional capacity and prevent disability. Routine cognitive screening should enable early detection of cognitive decline and facilitate timely, targeted interventions.

Access to and training in telemedicine should be promoted to support remote monitoring and continuity of care. These tools should also be made available to caregivers, thereby enhancing their capacity to assist and coordinate care effectively. Furthermore, when requested, access to social networks; email communication; and more broadly, the use of digital devices should be supported to help reduce both social and cognitive isolation.

Ultimately, the question is, above all, an ethical one: Should our attention be directed primarily toward the very elderly or the young? This dilemma recalls the debate in ancient Rome between the positions of Publius Terentius Afro, on the one hand, and the reflections of Cicero and Seneca, on the other, concerning the value of preserving the role of the elderly within society.

## Figures and Tables

**Table 1:** **AGING BIOLOGICAL PRINCIPLES** [3,4]—redrawn and modified in part from [3].

Cause/Principle	Description
Cellular and Molecular Damage	Accumulation of oxidative stress, DNA mutations, telomere shortening, and protein misfolding, leading to functional decline and aging.
Molecular Mechanistic Principle	Frequency of damage and failure of 100% maintenance/repair.
Genetic Programming	No fixed genetic “clock” for the lifespan. Apoptosis and aging-related genes exist but do not “cause” aging directly. Aging is not genetically programmed.
Species and Tissue Differences	Aging varies among species, individuals, tissues, and cell types. Different aging rates within and across organisms. Aging is heterogeneous at every biological level.
Entropy/Thermodynamic Limits	Biological systems tend toward disorder, and this makes long-term homeostasis unsustainable.

**Table 2:** Median age and proportion of population aged 80 years and over in selected countries (latest available estimates from World Population Prospects 2022) [9].

Country/Region	Median Age (years)	Population 80+ (%)
European Union (average)	44.4	6.1
Italy	48.4	7.6
Mexico	29.2	0.9
Israel	30.4	2.0
Philippines	25.7	0.47
India	28.2	0.75
Japan	48.6	10.0
China	39.0	2.4
United States	38.5	3.8
Brazil	34.2	2.1
